# Statins Increase the Frequency of Circulating CD4^+^FOXP3^+^ Regulatory T Cells in Healthy Individuals

**DOI:** 10.1155/2015/762506

**Published:** 2015-02-22

**Authors:** Ana Lucía Rodríguez-Perea, Carlos J. Montoya, Sven Olek, Claire A. Chougnet, Paula A. Velilla

**Affiliations:** ^1^Grupo Inmunovirología, Facultad de Medicina, Universidad de Antioquia (UdeA), Calle 70 No. 52-21, Medellín, Colombia; ^2^Epiontis GmbH, 12489 Berlin, Germany; ^3^Division of Immunobiology, Department of Pediatrics, Cincinnati Children's Hospital Research Foundation, Cincinnati, OH 45229, USA

## Abstract

Statins have been shown to modulate the number and the suppressive function of CD4^+^FOXP3^+^ T cells (Treg) in inflammatory conditions. However, it is not well established whether statin could also affect Treg in absence of inflammation. To address this question, eighteen normocholesterolemic male subjects were treated with lovastatin or atorvastatin daily for 45 days. The frequency and phenotype of circulating Treg were evaluated at days 0, 7, 30, and 45. mRNA levels of FOXP3, IDO, TGF-*β*, and IL-10 were measured in CD4^+^ T cells. We found that both statins significantly increased Treg frequency and FOXP3 mRNA levels at day 30. At day 45, Treg numbers returned to baseline values; however, TGF-*β* and FOXP3 mRNA levels remained high, accompanied by increased percentages of CTLA-4- and GITR-expressing Treg. Treg Ki-67 expression was decreased upon statin treatment. Treg frequency positively correlated with plasma levels of high-density lipoprotein cholesterol (HDL-c), suggesting a role for HDL-c in Treg homeostasis. Therefore, statins appear to have inflammation-independent immune-modulatory effects. Thus, the increase in Treg cells frequency likely contributes to immunomodulatory effect of statins, even in healthy individuals.

## 1. Introduction

Regulatory T cells (Treg) are a subpopulation of CD4^+^ T cells that control innate and adaptive immune responses [1]. These cells have two main origins: they are either thymus-derived or peripheral-derived (tTreg and pTreg, resp.) [2]. Regardless of their origin, Treg are characterized by high expression of the IL-2 receptor alpha chain (CD25), low expression of IL-7 receptor (CD127), and expression of the transcription factor FOXP3. Demethylation of the highly conserved CpG-enriched element, located in the 5′ untranslated region (5′UTR) of* FOXP3* called Treg cell-specific-demethylated-region (TSDR), is essential for Treg maintenance [3]. Because activated human conventional CD4^+^ T cells can also express CD25 and FOXP3 [4, 5], this epigenetic feature is considered as a more reliable marker to distinguish between activated T cells and real Treg [3].

Diverse effects of Treg cells have been observed, either beneficial or detrimental, depending on the clinical context. The beneficial role of Treg-mediated suppression has been established in different conditions, such as cardiovascular and cerebrovascular diseases, asthma, inflammatory diseases, allergy, autoimmune diseases, and graft-versus-host diseases [6, 7]. Therefore, increasing the frequency and/or function of Treg would be useful in patients suffering from these diseases if Treg-inducing drugs with a good safety profile and few toxic effects could be used. Statins are drugs traditionally used to reduce low-density lipoprotein cholesterol (LDL-c) levels, thus diminishing the risk for cardiocerebrovascular diseases. They act on the synthesis of cholesterol and isoprenoids, through inhibition of the enzyme 3-hydroxy-3-methyl-glutaryl-CoA reductase [8]. However, an immunomodulatory action of statins has also been demonstrated [8]. In particular, statin treatment was shown to increase Treg frequency and enhance their suppressor capacity, in subjects with hypercholesterolemia [9] and in patients with rheumatoid arthritis [10]. However, the direct effect of statins on the immune system is difficult to establish in these statin-treated patients with chronic inflammatory diseases due to multiple confounding factors. Furthermore, in these previous studies, Treg frequency was measured only before and after weeks of treatment, which does not give an in-depth picture of the dynamic of the Treg response to statin treatment. To address these gaps in knowledge, we studied Treg frequency and phenotype in healthy subjects treated with statins. Furthermore, Treg were studied at several time points following treatment initiation.

## 2. Materials and Methods

### 2.1. Study Design and Subjects

An experimental study was carried out in 21 healthy adult males over 18 years old. We excluded female volunteers due to potential variations in the number and function of Treg linked to hormonal variations [11]. In addition, epigenetic random X-inactivation affects the methylation status at the TSDR [3]. Included individuals had no history of chronic inflammatory diseases or hypercholesterolemia and had never taken statins.

Eighteen subjects received statins for 45 days, either 20 mg of atorvastatin (AV)/day (Biogen Laboratory, Bogotá, Colombia) or 40 mg of lovastatin (LV)/day (Laproff Laboratory, Medellín, Colombia). Blood samples were taken at days 0, 15, 30, and 45 of treatment. Serum levels of total cholesterol, triglycerides, and high-density lipoprotein cholesterol (HDL-c) were quantified by colorimetric assays. Aspartate and alanine hepatic transaminases were determined at days 0 and 45 of the statin treatment. In addition, we included a control group of 3 volunteers who did not take statins. This study was approved by the Bioethical Board for Human Research from the Universidad de Antioquia, Colombia, and written informed consent was obtained from all subjects before participating in the study.

### 2.2. Flow Cytometry Analyses of Treg

Heparinized whole blood was stained for 20 min with fluorochrome-labeled monoclonal antibodies against the following surface molecules: APC-Cy7-anti-CD3 (clone SK7, BD Pharmigen, San Diego, CA, USA), Pacific Blue-anti-CD4 (OKT4, eBioscience, San Diego, CA, USA), APC-anti-CD25 (BC96, eBioscience), Pe-Cy7-anti-CD127 (RDR5, eBioscience), FITC-anti-HLA-DR (L243, BD Pharmigen), and PE-anti-hGITR (110416, R&D SYSTEMS, Minneapolis, MN, USA). The cells were then fixed and permeabilized using the fixation/permeabilization buffer (eBiosciences) and stained for intracellular molecules using PerCP-Cy5.5-anti-FOXP3 (PCH101, eBioscience), PE-anti-CTLA-4 (14D3, ebioscience), and FITC-anti-Ki-67 (B56, BD Pharmigen). Appropriate isotype-matched control antibodies were included. FOXP3 positivity was defined in reference to the CD3^−^ population as previously described [12]. The samples were acquired on a FACSCanto II flow cytometer (Becton Dickinson (BD), Heidelberg, Germany), collecting a minimum of 100,000 events in the lymphocyte gate (defined by forward and side scatter parameters) and analyzed using the FACSDiva software (BD). Absolute numbers of Treg were calculated by multiplying the percentage of Treg by the absolute number of CD4^+^ T cells.

### 2.3. FOXP3 TSDR Methylation Analysis

In addition, genomic DNA was isolated from PBMCs using the DNeasy blood and tissue kit (Qiagen, Hilden, Germany) and the FOXP3 TSDR DNA methylation status was analyzed by Epiontis GmbH (Berlin, Germany) by TSDR-specific real-time PCR, as previously reported [13]. Hence, the number of Treg is expressed as percentage corresponding to the amount of TSDR demethylation in the* FOXP3* gene.

### 2.4. CD4^+^ T Cell Purification

PBMCs were isolated from heparinized blood by density gradient centrifugation (Ficoll-Hypaque, Sigma-Aldrich, St. Louis, MO, USA). Later, CD4^+^ T cells were enriched from approximately 1 × 10^7^ PBMCs, by negative selection, according to the manufacturer's instructions (CD4^+^ T Cell Isolation Kit II, human, Miltenyi Biotec, Bergisch Gladbach, Germany).

### 2.5. RNA Isolation and Real-Time PCR

Total RNA was extracted from 3 × 10^6^ CD4^+^ T cells using the RNAeasy Mini Kit (Qiagen), following the manufacturer's instructions. cDNA was synthesized from 1 *μ*g of RNA using the RevertAid First Strand cDNA Synthesis Kit (Thermo Scientific, Hanover, MD, USA). The cDNA obtained was diluted 1 : 4 and used in quantitative RT-PCR reactions using SYBR Green (qPCR Master Mix kit, Thermo Scientific). The following primers were used:* FOXP3*, Fwd: 5′-ACC TTC CCA AAT CCC AGT GC-3′ and Rv: 5′-CCT GGC AGT GCT TGA GGA AGT-3′;* TGF-β*, Fwd: 5′ CAG CAA CAA TTC CTG GCG ATA-3′ and Rv: 5′-AAG GCG AAA GCC CTC AAT TT-3′;* IL-10*, Fwd: 5′-GCT GAG AAC CAA GAC CCA GAC-3′ and Rv: 5′-GGA AGA AAT CGA TGA CAG CG-3′; indoleamine 2, 3-dioxygenase (*IDO*), Fwd: 5′-ACA GAA TGC TGG TGG AGG AC-3′ and Rv: 5′ GGA AGT TCC TGT GAG CTG GT-3′; and* ubiquitin* (UBQ) Fwd: 5′-CAC TTG GTC CTG CGC TTG A-3′ and Rv: 5′-CAA TTG GGA ATG CAA CAA CTT TAT-3′. The CFX96 Real-Time PCR Detection System (Biorad, Hercules, CA, USA) was used to obtain cycle threshold (Ct) values and the expression levels of target genes were normalized relative to the expression of UBQ as reference gene, using the equation 1.8−[^ΔC^], where 1.8 correspond to mean PCR efficiency of 80%. The data are expressed as relative units (RU).

### 2.6. Statistical Analysis

Baseline values were compared with each point time using Wilcoxon matched-pairs signed rank tests. Data are displayed as median and interquartile range (IQR). Correlations were tested by Spearman tests. In all tests, a *P* value lower than 0.05 was considered statistically significant. Statistical analysis was performed using GraphPad Prism v. 6.00 (San Diego, CA, USA).

## 3. Results/Discussion

### 3.1. Statins Affect Lipid Metabolism in Healthy Individuals

The median age (IQR) of the volunteers enrolled in this study was 26 [14–20] years. As expected, statin treatment significantly decreased total cholesterol, LDL-c, and triglyceride levels (Table 1). In contrast, HDL-c levels were significantly increased at day 45 of treatment (Table 1). Statins did not modify the plasma levels of hepatic transaminases in any of the study subjects (data not shown), suggesting that the intake of statins was safe and well tolerated. Of note, similar changes were found in LV- and AV-treated individuals (data not shown). None of the parameters evaluated significantly changed in the 3 volunteers who did not receive statins (data not shown).

### 3.2. Statins Increase Treg Frequency and Absolute Numbers in Healthy Subjects

Since the strategy to characterize human Treg by flow cytometry is still debated, we compared several marker combinations, namely, CD3^+^CD4^+^CD25^+^CD127^Low/−^, CD3^+^CD4^+^FOXP3^+^CD127^Low/−^, or CD3^+^CD4^+^FOXP3^+^. Percentages of CD25^+^CD127^Low/−^ cells were positively correlated with those of FOXP3^+^ (*r* = 0.71, *P* < 10^−5^). A positive correlation was also found for FOXP3^+^CD127^Low/−^ and FOXP3^+^ cell frequencies (*r* = 0.44, *P* < 10^−4^). We therefore used CD4^+^FOXP3^+^ as our definition of Treg.

**Figure 1:**
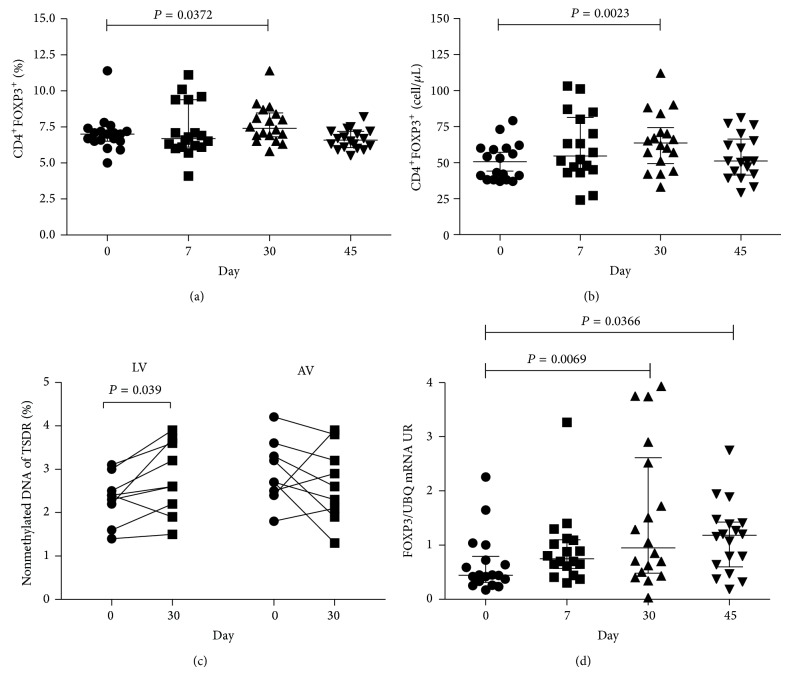
Statins increase the frequency of CD4^+^FOXP3^+^ Treg and mRNA FOXP3. The frequency (a) and absolute number (b) of CD4^+^FOXP3^+^ cells were evaluated by flow cytometry. Methylation percentage in the Treg cell-specific-demethylated-region (TSDR) of* FOXP3* gene, in DNA of PBMC from individuals before treatment and 30 days after ongoing treatment, is shown (c). FOXP3 mRNA expression relative to UBC housekeeping gene (d). Median, interquartile range (IQR), and *P* values are shown in the graph; difference between days was tested by Wilcoxon signed-rank test.

At day 30, statin treatment significantly increased the percentages of FOXP3^+^ cells within the CD4^+^ population, compared to baseline (*P* = 0.04, Figure 1(a)). Statins also increased absolute numbers of CD4^+^ T cells (1148 cells/*μ*L versus 959 cells/*μ*L, *P* = 0.007). In consequence, statins also increased Treg absolute numbers (*P* = 0.002, Figure 1(b)) at day 30, compared with day 0. These data are in agreement with previous studies showing that CD4^+^CD25^high^ T cells or CD4^+^CD25^+^FOXP3^+^ T cell frequency increases upon statin treatment in patients with inflammatory conditions [9, 10]. Interestingly, at day 45 of treatment, the peripheral Treg population returned to baseline numbers (Figures 1(a)-1(b)). A potential mechanism for this finding is that statins can modulate the expression of several chemokines and cell adhesion receptors by targeting the prenylation of small GTPases [21]. Such modulation might promote Treg migration to tissues [22]. Future studies will be needed to clarify this important issue.

We also evaluated at day 0 and day 30 the FOXP3 TSDR demethylation status in PBMC. No significant difference was found after treatment in the overall group (LV + AV). These data suggest that conversion of FOXP3^−^ T cells to FOXP3^+^ T cells may contribute to increased Treg numbers, as other authors have shown that atorvastatin, simvastatin, and lovastatin promote Treg conversion* in vitro* [9, 23]. However, in an independent analysis, LV treatment was found to increase the percentage of Treg with stable expression of FOXP3 at day 30 (2.8% versus 2.3%, *P* = 0.04), while no effect was seen in AV-treated subjects (Figure 1(c)). Mechanisms underlying this difference are not known, but it is possible that different statins may induce dissimilar epigenetic changes. Of note, a previous study showed that simvastatin, a statin similar to LV, can control the methylation of the* FOXP3* promoter region [23]. Furthermore, LV was shown to downregulate DNA methyltransferases (DNMTs), leading to the activation of several genes [24]. LV could thus induce epigenetic changes not only of* FOXP3* but also of other genes associated with Treg, such as* CTLA-4*,* GITR*, and* Eos*, whose expression is influenced by the hypomethylation of CpG islands [25]. Alternatively, AV may have a similar, but more modest effect on the FOXP3 TSDR methylation status, and our study might not have included enough individuals to detect these changes.

Enhanced transcriptional expression of FOXP3 by statins has been previously reported. The proposed mechanisms are associated with inhibition of geranylgeranylation, leading to the expression of SOCS3 (suppressor of cytokine signaling 3), an inhibitor of the IL-6/STAT-3 pathway, which tilts the differentiation of T cells towards Treg. In agreement with these hypotheses,* FOXP3* mRNA expression was higher at days 30 and 45 than that at baseline after treatment with statins (0.95 RU versus 0.44 RU, *P* = 0.007; and 1.18 RU versus 0.44 RU, *P* = 0.04, resp.; Figure 1(d)). Contrary to what was observed for FOXP3 TSDR demethylation status, similar changes in total FOXP3 mRNA were seen in LV- or AV-treated individuals.

### 3.3. Statins Change Treg Phenotype

We then determined whether statins modulated the mRNA expression of genes associated with Treg function, such as TGF-*β*,* IL-10*, and* IDO*. Levels of TGF-*β* transcripts in purified CD4^+^ T cells were elevated at day 45 compared to day 0 in treated individuals (0.27 RU versus 0.045 RU, *P* = 0.0007). In contrast, IL-10 and IDO mRNA expression did not significantly change in the treated individuals (data not shown). Increased TGF-*β* synthesis after statin treatment has been previously reported, which could also be mediated by the inhibition of geranylgeranylation [26, 27]. In addition, TGF-*β* could be involved in the statin-mediated changes of the phenotype and size of the Treg pool [28], as TGF-*β* promotes FOXP3 expression via Smad-dependent mechanisms [29].

**Figure 2:**
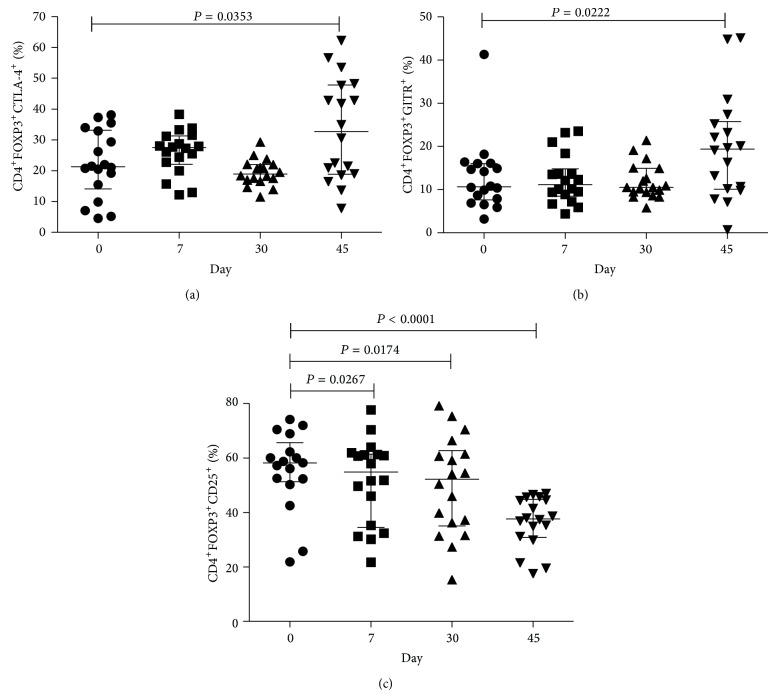
Statins increased the expression of Treg markers. Multiparameter flow cytometric analysis of percentage of CD4^+^FOXP3^+^ Treg expressing CTLA-4 (a), GITR (b), and CD25 (c). Median, interquartile range (IQR), and *P* values are shown in the graph; difference between days was tested by Wilcoxon signed-rank test.

The phenotype of circulating Treg has not been thoroughly examined in individuals treated with statins. We therefore evaluated by flow cytometry the expression of several markers associated with Treg activation, cell cycle, and suppressive function, such as HLA-DR, Ki-67, CTLA-4, GITR, and CD25. An increased proportion of FOXP3^+^ Treg expressing CTLA-4 (Figure 2(a)) and GITR (Figure 2(b)) were found at day 45 compared to baseline. No significant difference was found in the frequency of HLA-DR^+^ Treg (data not shown). Both* CTLA-4* and* GITR* genes are regulated by FOXP3 [14], which could explain their upregulation in statin treated individuals.

**Figure 3:**
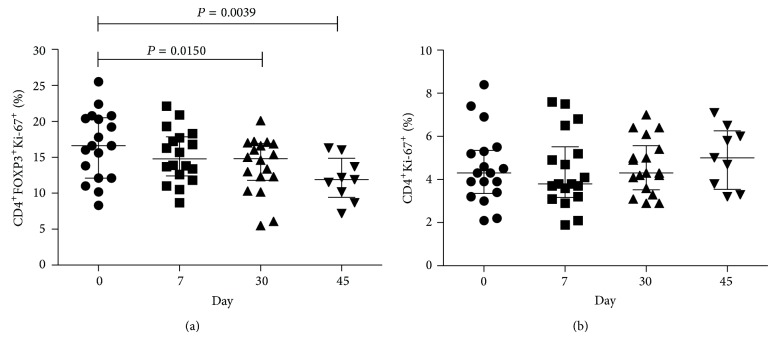
Treg from statins-treated individuals have lower Ki67 expression. Cell cycling was evaluated by the expression of Ki-67 by CD4^+^FOXP3^+^ Treg cells (a) and total CD4^+^ T cells from peripheral blood of healthy donors treated with statins (b). Median, interquartile range (IQR), and *P* values are shown in the graph; difference between days was tested by Wilcoxon signed-rank test.

In agreement with the previous studies showing that statins suppressed CD25 upregulation by T cells [15, 16], we also found decreased percentage of CD25^+^ cells within the CD4^+^FOXP3^+^ population at all time points compared to baseline (Figure 2(c)). We also examined Ki-67 expression by Treg because Ki-67 induction is a marker of cell activation/proliferation [17, 18]. We found reduced expression of Ki-67 by Treg in treated individuals, while it did not significantly change over time in total CD4^+^ T cells (Figures 3(a) and 3(b)). These data are in agreement with the fact that statins are known to inhibit cell proliferation [19]. Decreased CD25 and Ki-67 expression could be associated with the statin-mediated downregulation of the Ras-extracellular-signal-regulated-kinase (ERK) pathway [10], as this pathway is known to regulate CD25 expression and cellular proliferation [20, 30].

Taken together, these data thus suggest that augmented proliferation is not the mechanism underlying increased Treg frequency. In addition to augmented conversion, as proposed above, enhanced Treg survival could also be involved. Further studies will be needed to clarify this important point.

### 3.4. Treg Frequency Correlates with HDL Levels

**Figure 4:**
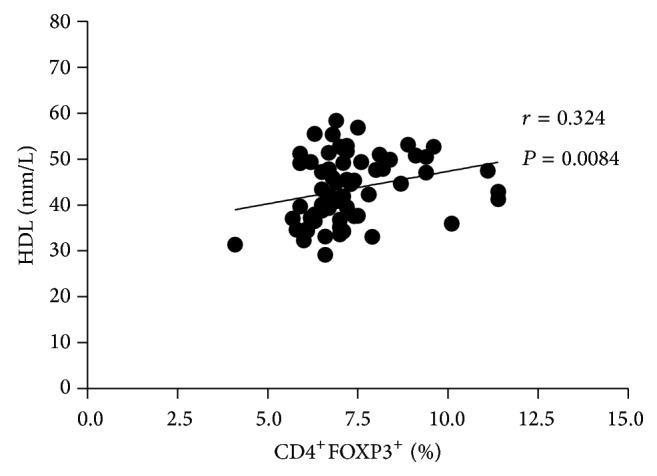
Increase in the HDL levels correlated with Treg frequency. Correlation of HDL levels with the percentage of Treg in the individuals of the study. Spearman coefficient (*r*) and *P* values are shown in the graph.

Reduction of cardiovascular risk by statins has been associated not only with diminished LDL-c levels but also with increased serum HDL-c levels. In addition to reverse cholesterol transport from the peripheral organs to the liver, HDL-c, along with its main apolipoprotein (ApoA-1), plays an anti-inflammatory role [31]. Indeed, ApoA-I has recently been shown in a murine model to increase Treg frequency, leading to decreased autoimmune responses [32]. We also found an increase of HDL-c levels after treatment with statins, mainly at day 45 (Table 1). Interestingly, HDL-c levels positively correlated with Treg frequency (*r* = 0.3245, *P* = 0.0084, Figure 4). Since HDL-c levels are negatively correlated with the frequency of proinflammatory T cell subsets [33] and anti-inflammatory and immunomodulatory properties of HDL-c have been widely reported [34], our results thus suggest that HDL-c could regulate Treg homeostasis. Interestingly, Treg were recently shown to directly affect fat metabolism and consequently modulate blood lipid levels, by regulating lipoprotein catabolism. Indeed, Treg depletion in murine models led to increased levels of large, cholesterol-rich, VLDL particles, due to their reduced clearance, and this effect appeared independent of vascular inflammation [35]. Altogether, these findings suggest that statin-induced Treg could also be beneficial in the context of atherosclerosis, due to the Treg control of hepatic fat metabolism.

## 4. Conclusion

Our results point to an effect of statins* in vivo* in noninflammatory situations/patients, affecting both the frequency and phenotype of the Treg subset, which could be associated with the increase in HDL-c levels. Our results confirm previous findings about the effect statins have on Treg in pathological settings characterized by uncontrolled inflammatory responses, such as hypercholesterolemia, rheumatoid arthritis, and acute coronary syndrome (reviewed in [36]). Importantly, the prescription of statins has been expanded and young adults are now candidates for statin therapy, regardless of their LDL-c levels [37]. Statins would be a safe and efficient way to boost Treg to prevent inflammation if their effect persists over time. However, the possible detrimental effects of Treg should also be considered, because increasing Treg frequency has the potential to dampen immune control of persisting infections, decrease vaccinal immune responses, and suppress antitumor immune responses, resulting in tumor cells escaping surveillance (reviewed in [38]). Therefore, additional studies will be necessary to understand the mechanisms underlying the effect of statins on Treg and their persistence in people treated for long periods of time.

## Figures and Tables

**Table 1:** Variation in serum lipids levels during the observational period.

Variable	Day 0	Day 7	Day 30	Day 45
Tc (mmol/L)	4.50 (4.14–4.94)	4.37^**^ (3.75–4.89)	3.57^***^ (3.13–4.32)	3.44^****^ (2.90–3.85)
LDL-c (mm/L)	2.48 (1.94–2.90)	1.78^**^ (1.39–2.17)	1.5^****^ (1.32–1.86)	1.83 (1.29–2.22)
HDL-c (mm/L)	1.06 (1.01–1.19)	0.98 (0.90–1.21)	1.16 (0.95–1.29)	1.16^*^ (1.01–1.32)
Tg (mm/L)	1.36 (1.0–2.06)	1.49 (1.16–1.71)	1.45 (1.01–2.01)	1.15^*^ (0.96–1.52)

Data are expressed as median (25th to 75th percentile). Tc: total cholesterol, LDL-c: low density lipoprotein cholesterol, HDL-c: high density lipoprotein cholesterol, Tg: triglycerides. ^*^ *P* < 0.05 as compared to baseline level. ^**^ *P* < 0.01 as compared to baseline level. ^***^ *P* < 0.001 as compared to baseline level. ^****^ *P* < 0.0001 as compared to baseline level.
